# Quality of life in age-related macular degeneration: a review of the literature

**DOI:** 10.1186/1477-7525-4-97

**Published:** 2006-12-21

**Authors:** Jan Mitchell, Clare Bradley

**Affiliations:** 1 Department of Psychology, Royal Holloway, University of London, Egham, Surrey, TW20 0EX, UK

## Abstract

**Background:**

The Age-related Macular Degeneration Alliance International commissioned a review of the literature on quality of life (QoL) in macular degeneration (MD) with a view to increasing awareness of MD, reducing its impact and improving services for people with MD worldwide.

**Method:**

A systematic review was conducted using electronic databases, conference proceedings and key journal hand search checks. The resulting 'White Paper' was posted on the AMD Alliance website and is reproduced here.

**Review:**

MD is a chronic, largely untreatable eye condition which leads to loss of central vision needed for tasks such as reading, watching TV, driving, recognising faces. It is the most common cause of blindness in the Western world. Shock of diagnosis, coupled with lack of information and support are a common experience. Incidence of depression is twice that found in the community-dwelling elderly, fuelled by functional decline and loss of leisure activities. Some people feel suicidal. MD threatens independence, especially when comorbidity exacerbates functional limitations. Rehabilitation, including low vision aid (LVA) provision and training, peer support and education, can improve functional and psychological outcomes but many people do not receive services likely to benefit them. Medical treatments, suitable for only a small minority of people with MD, can improve vision but most limit progress of MD, at least for a time, rather than cure. The White Paper considers difficulties associated with inappropriate use of health status measures and misinterpretation of utility values as QoL measures: evidence suggests they have poor validity in MD.

**Conclusion:**

There is considerable evidence for the major damage done to QoL by MD which is underestimated by health status and utility measures. Medical treatments are limited to a small proportion of people. However, much can be done to improve QoL by early diagnosis of MD with good communication of prognosis and continuing support. Support could include provision of LVAs, peer support, education and effective help in adjusting to MD. It is vital that appropriate measures of visual function and QoL be used in building a sound evidence base for the effectiveness of rehabilitation and treatment.

## 1. Age-related macular degeneration

Age-related macular degeneration (MD) is a chronic, progressive eye disorder that mainly affects people over the age of 50. It is the leading cause of blindness in the Western world in people over 60 yrs [1] and the third most common cause globally after cataract and glaucoma [1]. Data collated in 2002 indicated that MD was the cause of 8.7% of the global estimate of 161 million cases of visual impairment [2]. Recently it was estimated that, in the UK, with a population of 59 million [3], approximately 417,000 people have some degree of MD, of whom 214,000 have sufficient visual impairment for registration as partially sighted or blind [4]. Now that people are living longer the prevalence of MD is likely to increase [4].

MD leads to loss of central vision needed for activities requiring fine vision such as reading, driving and recognising faces. Peripheral vision is usually retained but MD can impair proficiency in performing most activities in daily living and can make it more difficult for people to live independent lives.

There are two types of MD. Dry MD (also called atrophic MD) accounts for about 80% of cases and generally develops slowly, often affecting both eyes simultaneously. It usually, but not always, causes only mild loss of vision. Dry MD is characterised by fatty deposits behind the retina which cause the macula to thin and dry out. Wet MD (also called neovascular MD) is associated with rapidly deteriorating vision and severe impairment and accounts for 90% of cases of severe visual impairment due to MD. Wet MD is caused by the growth of new blood vessels (a process known as choroidal neovascularisation (CNV)) behind the retina. These new blood vessels are weak and have a propensity to leak, damaging the retinal cells and leading to scar tissue. There are subtypes of wet MD, known as 'classic' and 'occult'. In classic CNV, the new blood vessels can be seen distinctly by an ophthalmologist using angiography. In occult CNV, the leaking vessels are obscured. Patients may present with a combination of both occult and classic CNV.

MD is a largely untreatable condition. Treatment is appropriate for a small percentage of people if they are diagnosed at an early stage with particular types of the wet form of the disease. Even then the treatment does not cure the condition but can limit its progress, at least for a time [5]. However, potential new treatments and rehabilitation interventions are continually being developed and tested.

This report critically reviews the literature that relates to the effects of MD on quality of life (QoL), and identifies strategies most likely to improve QoL for the many people who live with MD.

## 2. Measuring quality of life in MD

Although a great deal of QoL research is carried out there is little agreement about the definition of QoL. The one we prefer is based on the work of Joyce [6] and McGee and colleagues [7]:

"Quality of life is how good or bad you feel your life to be." [8]

Implicit in this definition is that QoL is a subjective perception and that QoL means different things to different people. Although many so-called quality of life measures allow people to indicate their own perceived levels of whatever aspect of life is being measured, many do not allow individuals to report the relevance or importance of that aspect of life for them [9-12]. Table 1 details the different measures referred to in this report that have been used to measure QoL and other patient reported outcomes (PROs) in research into MD.

**Table 1:** Patient reported outcomes referred to, constructs measured and populations for which they are validated

Measure	Country of origin	Actually measures						Validated for MD or closest population
		
		HS	LS	FS	VF	WB	QoL	
Hospital Anxiety and Depression Scale (HADS) [13]	Denmark					√		elderly
SF-36 [14]	USA	√				√*		elderly
W-BQ12 [15]	UK					√*		MD
HUI-3 [25]	Canada	√				√		community
EQ5D (EuroQol) [27]	Europe	√				√		community
Instrumental Activities of Daily Living scale (IADL) [30]	USA			√				MD
Sickness Impact Profile (SIP) [31]	UK			√		√		Elderly
Sickness Impact Profile for vision (SIPv) [32]	UK				√	√		Retinal disease
Activities of Daily Vision Scale (ADVS) [33]	USA				√			MD
14-item Vision Function Questionnaire (VF-14) [34]	USA				√			MD
Daily Living Tasks Dependent on Vision (DLTV) [36]	UK				√			MD, cataract
Low vision QoL (LVQOL) [37]	UK				√	√		mixed sample including MD
National Eye Institute Vision Function Questionnaire (NEI-VFQ25, 39, 51) [38]	USA				√	√		MD
Measure of the Impact of MD on QoL (MacDQoL) [42, 44]	UK						√	MD
Profile of Mood States (POMS) [65]	USA					√*		elderly
Quality of Well-being Scale (QWB) [66]	USA			√				elderly
Diagnostic and statistical manual (DSMIV) [69]	USA					√		adults
Functional Vision Screening Questionnaire (FVSQ) [71]	USA				√			Vision impaired
Community Disability Scale (CDS) [72]	USA			√				adults
Life Satisfaction Index – Wellbeing [87]	UK		√					elderly
General Health Questionnaire [110]	UK					√*		elderly
Positive and Negative Affect Scale [131]	USA					√*		adults
Vision-related QoL (VQOL) [117]	UK				√	√		Vision impaired
Manchester Low Vision Questionnaire (MLVQ) [118]	UK				√			MD
Geriatric Depression Scale [115]	UK					√		elderly
Psychological General Well-being Index (PGWB) [123]	USA					√*		adults
Multilevel Assessment Instrument (MAI) [132]	USA			√				elderly
Freiburg Inventory on Coping with Illness [133]	Germany	coping						adults

* indicates that both negative and positive well-being are measured

### 2.1 Measuring Patient Reported Outcomes (PROs) in eye disease

#### 2.1.1 Psychological well-being measures

These measure mood. People who feel depressed and anxious are unlikely to describe their QoL as good. However, even those who are not depressed or anxious may still feel that their QoL is severely damaged by MD. Some well-being scales, such as the Hospital Anxiety and Depression Scale (HADS) [13] measure only negative well-being (anxiety and depression). Where people have no anxiety or depression to begin with, such a measure could show no improvement. Measures which also investigate positive well-being (e.g. the well-being scales within the SF-36 measure vitality [14]) and particularly those which measure positive well-being with items concerned with enthusiasm for life (e.g. the Well-being Questionnaire (W-BQ12) which measures energy and positive well-being as well as anxiety and depression [15]) are more likely to detect improvement in psychological well-being.

#### 2.1.2 Health status (HS) measures

These investigate subjective perceptions of health but unfortunately HS measures are often wrongly called QoL measures and this has caused great confusion and misleading conclusions [11]. HS is not QoL, although poor HS may be associated with impaired QoL. Good HS does not indicate that QoL is good. HS measures are rarely any use as indicators of the impact of eye conditions on QoL because most of the domains investigated are not affected by visual impairment (e.g. pain, energy, appetite). For example, the SF-36 [14], a generic HS measure, and the shorter subset, SF-12, have been found to be sensitive to age-related eye disease including MD in some work [16] but not in most studies [17-21] and found to be only minimally responsive to change in visual acuity (VA) in patients with CNV, over a period of 2 years [22]. It was not responsive to the impact of low vision services [23]. The SF-12 was also found not to be responsive to change [24]. The health utility index (HUI-3) [25] includes items concerned with vision and, unsurprisingly, proved more sensitive to vision impairment [26] than the SF-12 and the EQ5D [27] which investigates only five dimensions of health, none of which is vision. This disappointing performance of widely used HS measures in detailing impairment in people with MD and other eye conditions can be understood when it is appreciated that, for the most part, the general population do not think of problems with their eyesight when asked about their health. Patients maybe registered blind with MD and still report that their health is excellent. If asked about their QoL they may nevertheless say it is shot to pieces by their MD. Quality of health is quite a different matter from quality of life [11] and this is particularly true for people with eye conditions, including people with MD. When the SF-36 or EQ5D show no impact of MD and are also wrongly referred to as QoL measures it may be mistakenly concluded that MD has no perceived impact on QoL when all that has been shown is that MD has no perceived impact on health. The literature on MD abounds with studies that have used health status measures and wrongly referred to these as QoL measures (e.g. [21,24,28,29]). It is essential that we recognise this problem and are not misled by the data.

#### 2.1.3 Functional status (FS) measures

These questionnaires investigate respondents' ability to carry out activities of daily living (ADL) such as self-care and eating. They do not specifically investigate vision-related activities (e.g. reading, watching TV), although they may contain some items that are relevant to vision. Often they do not include psychological domains such as confidence or worry. They do not necessarily correlate well with objective measures of vision or with QoL because FS measures only ask what a person can do, not whether they want or need to do those things or how important they are to their QoL. Nevertheless, using the Instrumental Activities of Daily Living (IADL) scale, designed for the study, Williams et al [30] demonstrated that, compared to visually unimpaired elderly people, patients with MD were 8 times more likely to report difficulty shopping, 13 times more likely to have difficulty managing finances, 4 times more likely to experience difficulties preparing meals, 12 times more likely to have problems using a telephone, and 9 times more likely to experience problems with light housework. The Sickness Impact Profile (SIP) [31] is a measure of health-related disability. A vision-related version of the scale (SIPV) [32] was developed in which, for each question from the SIP answered affirmatively, indicating dysfunction, a subsequent question asks if visual dysfunction contributes to the reported difficulty. An American study of 86 retinal patients (not elderly) found that SIPV scores showed increased disability across mild, moderate and severe visual impairment and all but one of the SIP subscales demonstrated greater disability in patients than controls [32]. The SIP and SIPV were highly correlated but, in this study, over half the participants had diabetic retinopathy and disability due to other diabetes-related complications may well have influenced the result.

#### 2.1.4 Vision-specific functional status (VF)

These measures investigate vision-related tasks such as reading, writing, watching TV, recognising faces or driving. They are usually correlated with standard measures of vision such as VA. However, because they do not differentiate between what is relevant and what is irrelevant to individual respondents, or what is important to QoL and what is not, they are not true QoL measures, although they are frequently referred to as such [10,12]. The impact on QoL of loss of or deteriorating near vision would be greater for someone who spent a lot of time reading and doing embroidery than for someone who preferred listening to music and swimming. VF measurement has also been shown to be influenced by general health [17]. For example, the ability to prepare a meal may be affected by arthritis as well as by vision and, if the questionnaire does not specifically ask the respondent to consider only the effects of their vision on a task, co-morbidity may confound the scores and make results difficult to interpret. The Activities of Daily Vision Scale (ADVS) [33] was found to discriminate between mild and severe MD (overall score, near vision, daytime driving and glare) but not between mild and moderate MD [29]. The VF-14 [34], originally designed for use with cataract patients correlated more highly with patients' global assessments of their vision than did VA in a Canadian study of 159 MD patients [18] but MD severity did not predict VF-14 scores. Since the VF-14 scores and patients' global assessments of their vision are both effectively self-reports of vision function, it is not surprising that they correlated with each other better than with a more objective measure of VA or a measure of MD severity based on clinical features of the eye. A Finnish study [35] reported similar properties for the VF-14. A visual function measure designed for use with MD and cataract patients is the Daily Living Tasks Dependent on Vision (DLTV) questionnaire [36]. It was found to correlate more strongly with distance VA in the better eye than in the worse eye.

The 3 measures discussed above (ADVS, VF-14, and DLTV) investigate only visual function and do not include items relating to social or psychological functioning. The Low Vision Quality of Life questionnaire (LVQOL) [37] was designed for the evaluation of low vision rehabilitation and does include a 3-item subscale relating to adjustment. In a sample of 278 patients and 70 matched controls, the measure differentiated between people with normal vision and people with low vision. LVQOL scores improved by an average of 17% following rehabilitation, compared with people with normal vision, with reading and fine work subscales most improved, but psychological adjustment and other subscales also showed improvement [37]. The NEI-VFQ [38], which has been well validated in the MD population also investigates psychological aspects of visual impairment. As well as items pertaining strictly to function, the NEI-VFQ investigates social functioning, mental health and dependency. It differentiates between different eye conditions and overall score and relevant subscale scores are correlated with VA [38]. It has been shown to be responsive to change in VA over time [39], but this was in a large study over a long period of time and the minimum change in VA investigated was 3 lines of vision. A 3-line vision loss has been used as the primary outcome measure in clinical trials for treatments for MD (e.g. [40]). Two lines has also been recommended as a clinically significant change [41]. A measure may not be responsive in smaller samples over a shorter period of time, when less change might be expected to take place, if it cannot detect a change of only two lines. It remains to be seen if the NEI-VFQ is sufficiently responsive to detect two-line vision loss.

#### 2.1.5 Vision-specific individualised quality of life measures

These investigate the impact of vision impairment on QoL, examining both impact and importance of each domain on QoL and allowing for variability in the relevance of specific domains to individual respondents [42,43]. Impact and importance scores are multiplied to give weighted impact scores. The MacDQoL [42,44] measure of the impact of MD on QoL has two overview items (present QoL and MD-specific QoL) and 23 domain-specific items. It has been shown to differentiate between mild and moderate and mild and severe MD (measured by UK registration status: blind, partially-sighted or not registered) but, in common with visual function measures, not between moderate and severe. The overview items are also sensitive to severity of MD, the present QoL (generic) item less so than the MD-specific item, as would be expected. There are promising indications of the MacDQoL's responsiveness to change in a small sample (Mitchell, Wolffsohn, Woodcock et al, manuscript in preparation), and this warrants investigation in a larger study. Measuring both the impact and the importance of a domain of life to QoL leads to considerable variability in scores and so correlations between the MacDQoL and measures of vision such as VA or contrast sensitivity may not be as large as those between VA and visual function e.g. MacDQoL average weighted impact score correlates 0.45 with better eye distance VA [42], NEI-VFQ distance vision score correlates 0.65 with better eye distance VA [38] This latter correlation is perhaps not surprising since, for the NEI-VFQ score and the distance VA score the patient is being asked how well he or she can see in both cases. The MacDQoL measure captures the nature of the impact of MD on a person's life in a way that cannot be achieved with a vision function measure. Any loss of sensitivity to change in VA is outweighed by the increased relevance of the QoL measure to the whole experience of MD including experience of any treatment and rehabilitation.

### 2.2 Validation of questionnaires

Measures of health status, functional status, visual function and well-being are not, in themselves, QoL measures. However, they are all concerned with aspects of life that may be important to QoL.

The value of questionnaire data collected depends on the psychometric properties of the measure. Psychometric properties that are regarded as important in a measure include [10]:

• Content validity: The extent to which the topic of interest is comprehensively and relevantly investigated by the measure. Patient involvement in the design of a patient reported outcome measure is vital in ensuring content validity.

• Face validity: The extent to which the questionnaire appears to measure what it is intended to measure. Researchers selecting questionnaires should consider the questions carefully. They would then see that the EQ5D (also called EuroQol) in fact asks about health and not about QoL [11].

• Internal consistency reliability: The extent to which the items contribute to measuring the same construct (a reliability coefficient is calculated).

• Test-retest reliability: The extent to which scores remain stable over time when no change has occurred (i.e. when there has been no change in vision, no treatment for MD or rehabilitation).

• Construct validity: Hypotheses concerning the relationship of questionnaire scores to other measures (such as VA or contrast sensitivity) are tested. Ability to discriminate between levels of disease severity (e.g. between people who are registered blind, partially sighted or not registered) is important, particularly for a visual function measure, which would be expected to correlate strongly with disease severity.

• Responsiveness: Sensitivity to real change over time (e.g. deterioration in VA or contrast sensitivity).

• Interpretability: The extent to which change scores can be interpreted and explained.

In addition to these psychometric properties, the burden placed upon respondents should be considered (length of questionnaire, complexity of language, relevance of the questions) and that on administrators [45]. Where questionnaires are designed in one language and translated into other languages, linguistic validation is required, including cultural adaptation where needed [46]. Forward and backward translations are necessary (preferably reviewed by the questionnaire author) to ensure that the translations have not introduced semantic discrepancies. Clinician review can be helpful and cognitive debriefing interviews with people who have MD are needed to ensure that the translated items and instructions are understood as intended. Psychometric evaluation of each language version is necessary, at least on first use, before analysing data from multiple languages as one dataset.

The method of administration is a further consideration that is particularly important in visually impaired populations. Self-completion (pen and paper) has been found to elicit poorer scores than interview administration in some questionnaires [47-49] but not in others [33]. Where scores differ, using two implementation methods in one study may result in people with worse vision, and having interview administration, under-reporting impairment compared with people self-completing the measure. This would confound the results. Generally it is better to use only one administration method in any one study.

### 2.3 QALYs and other manipulations of PROs

A limitation of condition-specific or vision-specific measures of health status, functional status and QoL, even when they are interpreted appropriately, is that the scores are not comparable across diverse medical specialties [50]. One use for outcome measures is to assess the relative cost-effectiveness of different treatments and to inform decisions concerning allocation of limited funds. Such a measure, that could be used across all medical conditions and allow direct comparison, would be an asset for health economists. One technique that is adopted increasingly to achieve such comparisons is utility assessment. Utility values (also called preference measures) are quantitative expressions of preference for given health states. A scale is used where utility values range from 0 to 1; 0 represents death and 1 represents perfect health. Techniques used for eliciting this value include time trade-off (TTO) and standard gamble (SG) (see below). The utility value obtained can be used in conjunction with an estimate of life expectancy to calculate Quality Adjusted Life Years (QALYs). QALYs are estimates of life expectancy in full health. One year of life in a health state rated as perfect health (utility value of 1) = 1 QALY. Two years of life in a health state with a utility value of 0.5 = 1 QALY. Using a figure for the cost of treatment, a cost per QALY can be calculated. Such costs can be calculated for any clinical intervention, and have been used by medical decision makers such as the National Institute for Health and Clinical Excellence (NICE) in the UK to make choices between treatments. Health economists argue that non-preference PROs correlate poorly with preference measures and so are not suitable for use in economic evaluation [51]. It could be argued that preference measures, although convenient for calculating QALYs, do not correlate well with non-preference based measures as they do not measure quality of life [52]. Evidence that this is the case will be reviewed below. First, methods of obtaining utility values will be considered.

#### 2.3.1 Obtaining utility or preference measures

##### Time trade off

Participants are asked first how many more years they expect to live. They are then asked how many years of their remaining life they would be willing to give up if they could have a (hypothetical) treatment for their medical condition that would guarantee them perfect health for their remaining years. For example, if someone expected to have 20 years of life remaining, and were willing to give up 10 years in return for perfect health, then that person would be said to have given their medical condition a utility of 0.5.

##### Standard gamble

Participants consider two alternatives a) a treatment with two possible outcomes: either perfect health for the remainder of their life or immediate death b) the certainty of a chronic health state for life. Participants are asked to say what percentage risk of death they would be prepared to accept to avoid the certainty of having the chronic health state for the rest of their lives. Again the data are used to obtain a utility value of between 0 and 1.

A number of studies by a US research group have reported utility values for MD using TTO or SG techniques [53-58] and there has been reasonable concordance in the findings. A British study reported that utility values were more strongly associated with contrast sensitivity than VA [26]. Nevertheless the method has attracted criticism. For example, there is some debate about whose values are the most appropriate: patients', doctors' or those of the taxpaying general public [59]. The general public may be unaware of the impact of some medical conditions unless they themselves are affected by the condition. There can be marked differences in the values of patients, doctors and the public [60] and the decision to use one group rather than another will therefore be likely to affect the results obtained. It has also been reported that demographic data may be more predictive in determining health state utilities than the health states themselves [61]. Some studies have reported less than impressive response rates [62,63]. Others have avoided reporting response rates (e.g. [53,54]). TTO questions are often posed to patients during an eye clinic appointment while they wait to see the ophthalmologist following dilation of the pupils. Patients may feel vulnerable and disempowered at this time and reluctant to express unwillingness to take part. When participants in a UK study were asked TTO questions during a telephone interview while they were in their own home, at a time convenient to them, response rates were a cause for concern [52]. A large proportion of people who did respond (38%) said they would trade no time for perfect vision. Unsolicited comments from participants indicated that they thought the questions ridiculous, too hypothetical or objectionable for religious or other reasons. People said they would not trade time because they were carers or because they wanted to see their grandchildren grow up. Nevertheless, it is likely that improvement in their MD would improve their QoL. There was no relationship between utility values and vision status (registration as blind, partially sighted or not registered) whereas, in the same study, vision status was significantly associated with MacDQoL scores. Another UK study [64] demonstrated that 50% of participants with varying severity of MD were not prepared to trade any time for perfect vision and, after removing scores where no time was traded, there was no relationship between TTO utility values and VA. It is likely that the questions posed in the TTO method would be particularly difficult for elderly people to answer given their shorter life expectancies. The comparability of TTO responses to questions about 'perfect health' and those referring to 'perfect vision' must also be questioned. A person with poor vision and poor general health might view things differently from a person who has poor vision but otherwise good health.

Opinions differ as to whether utility values should be obtained from patients, health professionals or the tax-paying general public [51]. Generally, the public overestimate the impact of medical conditions on QoL compared with patients [9]. However it has been shown that MD is an exception to this rule: both the public and health professionals report higher utility values for MD than do patients [60]. This perhaps reflects an underestimation of the impact of the loss of central vision and an overestimation of the value of peripheral vision. Whatever the reason, a comparison of utility values across diseases when the utilities have been obtained from the public would mitigate against resources being allocated for treatment and rehabilitation of people who have MD.

## 3. Impact of MD

### 3.1 Psychological well-being

A number of studies have looked at the impact of MD on psychological well-being. In an American cross-sectional study investigating 86 MD patients with a VA of 20/200 or worse in at least one eye [30] participants reported greater emotional distress (Profile of Mood States [POMS] [65]) than similar aged people without visual impairment. Scores were comparable with those of people with serious illnesses such as melanoma and HIV. Poorer functional status (QWB [66]) was associated with greater emotional distress. Longer duration of MD was associated with lower levels of emotional distress, probably due to adaptation to the condition. Lack of adaptation, particularly the personal experience of vision loss as opposed to its effect on relationships, was associated with depression in an American study of 144 MD patients [67].

A further US cross-sectional study of 151 patients with advanced MD (VA of 20/60 or worse in the better eye) [68] reported that the rate of depressive disorder (32.5%) (DSM IV [69]) was twice that found generally among elderly people living in the community. The strongest associations found were between depression (DSM IV [69]) and both vision-specific (NEI-VFQ [38], SIPv [32]) and general disability (SIP [31]). There was only a weak association between VA and depression. In cross-sectional studies there is a potential reciprocal relationship between visual disability and depression (disability leads to depression and depression influences disability) [70], but in a longitudinal study causal effects in such relationships can be established.

A prospective, longitudinal US study recruited MD patients with recent (6 weeks previously) loss of vision in their second eye [70]. At baseline 17 (33%) of 51 participants met the criteria for clinical depression (a higher rate than the 16% found in the community) of whom only one was receiving treatment for depression, suggesting low levels of pre-existing depression. This group had poorer VA, and greater visual disability (Functional Vision Screening Questionnaire [FVSQ] [71]) and general disability (Community Disability Scale [CDS] [72]) than the non-depressed group. Six months later 40 people were followed up (people who dropped out tended to be more depressed at baseline than the follow-up participants). In people depressed at baseline and still depressed at follow-up (N = 7), those whose depression worsened had a corresponding decline in general and visual function independently of any change in VA.

Visual impairment in the elderly has been shown to be a predictor of suicide [73].

Decline in visual function inevitably leads to difficulties in performing daily activities and also in pursuing leisure activities. In a US study of 51 MD patients [74], 36 reported that they had lost valued activities as a result of impaired vision, reading and driving being the most common. In this sample, the relationship between VA and depression was mediated by loss of valued activities. In a postal survey of 2000 members of the UK Macular Disease Society using the Macular Disease Society Questionnaire (MDSQ) (Mitchell J, PhD thesis, Royal Holloway, University of London 2003) [75], 832 of 1420 respondents reported a reduction in the number of hobbies pursued due to vision loss since the onset of MD. In the entire sample the mean number of hobbies reported before MD was 3.54 compared with 2.20 at the time of the survey. Reading, enjoyed by 59% before diagnosis, but by only 20% at the time of the survey, was the most curtailed hobby, and crafts and driving were also severely affected. Poorer *Well-being *(W-BQ12), *Present QoL *and *MD-specific QoL *(MacDQoL overview items) were all associated with loss of leisure activities after controlling for severity of MD (registration status). Data from the MacDQoL [42,44] indicate that the domain of leisure activities is one of the most highly negatively impacted by MD.

Impaired efficiency in carrying out activities of daily life can compromise cherished independence [76]. In a study of 156 people with MD, independence was the most highly negatively impacted domain of the MacDQoL [42]. An Australian qualitative study [77] reported that losing the ability to drive is a major factor in loss of independence and, as well as having to rely on others for mobility, can lead to social isolation, in itself a factor in the progress of depression. This study also indicated that people resist asking for help for fear of becoming a burden and go to great lengths to remain independent.

Loss of independence in MD may be such that it is necessary to go into residential care. Visually impaired people are over-represented in care homes [78-80] where it is associated with elevated levels of depression [81]. Chalifoux [82] asserted that people with MD may be pressured by their families into entering residential care for their own safety, but he recommended that they should be encouraged to remain in the community for as long as it is practically possible.

Visual impairment due to MD may lead to unplanned for expense. In this elderly population, many of whom have very limited resources, financial outlay related to vision impairment may lead to hardship. Nevertheless, a French study found that people with MD pay for help in the home [83] and that expenses increased with worse VA. British research indicates that people spend money on optical and other devices [84]. Tasks such as home maintenance and dressmaking may have to be paid for where, prior to MD, people were able to carry out the work themselves. Some forms of treatment, such as photodynamic therapy (PDT), are expensive and may have to be paid for personally if National Health Service or insurance schemes will not allow the treatment. Treatments for conditions resulting from MD, including depression and fractures due to falls, may also be expensive [85]. 'Finances' was one of the least impacted domains of the MacDQoL [42,44] but even so there was a good deal of variability in the scores of both impact and importance on the finances domain, showing that, for some people, finances are important for QoL and are negatively impacted by MD.

There is a positive association between increasing age and poorer sleep quality [86]. Sleep disturbance is itself associated with depression and other somatic symptoms and causes a deterioration in quality of life [86]. In a study of 6,143 elderly Scandinavian people, Asplund found that poor sleep, difficulty falling asleep and frequent awakenings were all more common among visually impaired men and women than in those without visual impairment [86]. It is possible, therefore, that the sleep deprivation experienced by people with MD may contribute to elevated levels of depression.

### 3.2 Life satisfaction

Only one study was found that reported the relationship between life satisfaction and MD. A small Australian cross-sectional study reported that people with MD (N = 30) reported poorer life satisfaction (Life Satisfaction Index – well-being) [87] social support and greater stress due to daily hassles than a control group without MD of similar age [88].

## 4. Perceived quality of health care, satisfaction with the diagnostic consultation and their relationship to patient well-being

In the MDSQ study [75], 1420 respondents answered questions about their experiences at diagnosis. More than half, 735 (54%) thought their consultant was not interested in them as a person and 41% were dissatisfied with their diagnostic consultation. The attitude of the consultant and lack of information were the most frequently cited reasons for dissatisfaction (each cited by 43%). W-BQ12 scores were significantly poorer for those who were dissatisfied and for those who thought their consultants were not interested in them. In this cross-sectional study causality cannot be established but other data from the questionnaire added weight to the suggestion of a causal relationship. For example, the difference in well-being between the satisfied and dissatisfied groups was greater in more recently diagnosed people (< 2 years) than in those who had had MD for longer (Mitchell, 2003).

During the recent development of a measure of macular service satisfaction (MacSSQ) [89], items relating to aspects of the diagnostic consultation including provision of information, advice and support and the opportunity to ask questions were those that were most consistently rated as unsatisfactory by people taking part in focus groups and pilot work. Wong et al [77] said that the most striking feature of in-depth interviews in a qualitative Australian study of people with MD relating to the participants' psychosocial well-being was the importance of 'understanding' the condition. A focus group study in Sweden [90] demonstrated that people with MD wanted more information about many aspects of their condition and its consequences, including how they might prepare for a future with severe visual impairment.

The majority of MDSQ respondents (1247 (90%)) had been told that 'nothing can be done to help with your MD'. Of those 757 (61%) said they felt depressed or anxious on hearing this news and 54 (4.3%) said it led them to feel suicidal. Similar experiences at diagnosis were reported by MD patients in Australia [77].

The Royal College of Ophthalmologists' guidelines for the management of MD [91] stress the importance of early provision of information and support. Future research is needed to assess the extent to which the recommendations have been effective as far as the patients are concerned.

## 5 Extent of impairment

### 5.1 Bilateral and unilateral involvement

Williams et al [30] compared people with one or both eyes affected by MD in the US and found that those who were legally blind in only one eye reported greater emotional distress than those with bilateral blindness. Anecdotal evidence of greater distress among people with some vision in one eye compared with those with no vision was reported in much earlier work [92]. In both articles, fear of losing vision in the second eye was suggested as the cause of the greater distress. In contrast, in the MDSQ survey (Mitchell, 2003) respondents with only one eye affected reported better psychological well-being (W-BQ12) than those with bilateral MD. A qualitative study of men and women with varying severity of MD [77] found that people with only one eye affected reported that MD did not affect their day to day living but they worried about the future and the possibility of second eye involvement.

### 5.2 Dual sensory impairment

Many people experience deterioration in their hearing as they get older. When this is accompanied by visual impairment there can be considerable impact on QoL. Three population studies investigating dual sensory impairment in the elderly [93-95] reported that both vision and hearing impairment impacted negatively on FS, with visual impairment having the greater effect. These studies had only small numbers of participants in some of the impairment categories and relatively blunt measures of impairment were used but nevertheless they were able to report that dual sensory impairment had a greater impact than either visual or hearing impairment alone [93,94]. A large, longitudinal population study of older women (N = 6112) [96] reported that vision impairment alone, but not hearing impairment alone was associated with both functional and cognitive decline and women with dual sensory impairment were at greatest risk of functional and cognitive decline. Another population study of elderly people [97] relied on self-report of both vision and hearing impairment and a number of symptoms of depression. In spite of these rather crude measures, it was found that all three sensory loss groups (vision, hearing and dual) were more likely to report depressive symptoms than those without impairment, with greatest risk in the dual impairment group.

### 5.3 Co-morbidity

In studies of elderly populations it is likely that many people will have other medical conditions in addition to MD. Williams et al [30] reported that 66 of 78 participants with MD had other medical conditions. When compared with those in the sample who had MD as their only medical problem, there was no difference between the two groups in emotional distress, difficulties with IADLs, self-reported general health and functional status (QWB). Only 18% rated another of their medical conditions as worse than MD – a point that health economists would do well to take note of. A large, multinational study (Canada, France, Germany, Spain, UK) [98] found that neovascular MD patients had significantly more co-morbid conditions than a control group (2.5 vs 2.2) and the MD patients were more likely to have had cancer (9% vs 4%) and stroke (4% vs 1%). Falls were twice as common in the MD group (17% vs 8%) [85]. Conditions associated with age such as arthritis and osteoporosis can, for people with MD, further compromise their dexterity and confidence in carrying out daily activities, their ability to get out and about or to enjoy leisure activities and the effect may well be to reduce their QoL.

MD can also be the cause of co-morbidity. Hip fracture is common in the frail elderly population and visually impaired people are over-represented in this group of patients [99-101]. Dutton commented that a familiar experience for ophthalmologists is to send away a newly diagnosed woman with MD without follow-up or referral to other services because her VA does not warrant it only to have her return to the clinic some months later with a much reduced VA and a consequent fractured hip which has resulted in her being taken into residential care [102].

A commonly co-occurring eye condition for people with MD is cataract. The presence of MD has been implicated in poor visual outcome following cataract surgery and some evidence suggests that the treatment may even worsen the progress of MD [103]. Several studies, however, have indicated that cataract surgery leads to improved vision function for MD patients [103-105].

There is evidence to indicate that visual impairment carries with it an increased risk of mortality. McCarty et al [106] reported that VA worse than 6/12 more than doubled the risk of mortality over a 5-year period. The increased risk may be due to incidents such as falls or car accidents [106]. People with visual impairment may also be more prone to accidents in the home such as fires or mishaps with electrical appliances.

### 5.4. Visual hallucinations: Charles Bonnet Syndrome

Visual hallucinations, known as Charles Bonnet Syndrome, are common in people with visual impairment. In a survey of eye clinic patients with MD (N = 100), 13% reported experiencing formed visual hallucinations [107]. Only five of these had reported the hallucinations to their doctors. Of 86 consecutive patients at a retinal clinic (24% with MD), 13 (15%) said they experienced visual hallucinations [108]. Two of those participants had reported the hallucinations to a doctor. Two hundred and eighty two (20%) of the 1420 respondents to the MDSQ said they had experienced hallucinations at some time since diagnosis with MD [75]. Over 40% (122) of these had spoken to a health professional about the hallucinations of whom 59 (21% of those experiencing hallucinations) were given an explanation. Some explanations were inaccurate or unhelpful e.g. psychological (N = 1), brain confusion (N = 1), stress (N = 2), nothing to do with MD (N = 1). In a study of 100 patients treated with PDT for predominantly classic CNV, five (5%) described experiencing structured hallucinations following treatment [109].

These studies indicate that people may be reluctant to report hallucinations for fear of being diagnosed with dementia or otherwise considered 'crazy' [75,108]. This concern alone may impact on QoL, severity of MD and hallucinations notwithstanding. Mitchell reported that hallucinations were associated with poorer psychological well-being (W-BQ12 [15]) after controlling for severity of MD (Mitchell, 2003). Scott reported that, even among people with relatively good VA, those experiencing hallucinations demonstrated higher levels of emotional distress (General Health Questionnaire [GHQ] [110]) and decreased function (SIP [31]) compared with those without hallucinations [108]. Health professionals need to be aware of the likelihood of hallucinations in MD and ask patients if they have experienced them. Reassurance about the benign nature of the phenomenon and its often limited duration would prevent much distress [111,112]. Cohen et al [109] suggested that all patients who undergo PDT should be told beforehand that hallucinations are a possible side effect of treatment. Such pre-emptive education may be the best approach for all people diagnosed with MD [89].

## 6. Rehabilitation

People with MD may not understand the nature of the condition and consider it to be part of the ageing process [90]. If rehabilitation is to be successful, it is important that they appreciate that it is a medical condition, with disabling effects, many of which can be prevented or reduced [90] and that help is available.

The emotional impact of vision impairment is frequently discussed in terms of the 'loss model' [113]. According to this model vision loss is akin to bereavement and the patient will usually go through the stages of mourning, including shock, denial, anger and depression (which may take months) before rehabilitation can be effective. Dodds [114], however, asserted that depending on external support during depression may lead to helplessness that would later mitigate against effective rehabilitation and that the patient's own perceived incompetence resulting from a lack of early rehabilitation would lead to demoralization and depression. He recommended early intervention.

### 6.1 Low vision aids provision and training

Low vision services, including assessment of vision and of patients' needs, LVA provision and training have been shown to lead to improved visual function (VF-14, four subscales of NEI-VFQ) [23] and to lead to better visual function compared with a control group (LVQOL) [37]. Better psychological status (less depression and more self-confidence (Geriatric Depression Scale [GDS] [115]) at the time of rehabilitation has been shown to be associated with better outcome (reading speed and accuracy, critical print size) [116]. An interdisciplinary service including contributions from optometrists, ophthalmic nurses, social workers and rehabilitation officers resulted in improvements to visual function (VQOL [117]) and fewer problems in daily living (Manchester Low Vision Questionnaire [MLVQ] [118]) [119]. One study by Reeves et al compared standard low vision care with enhanced regimens but found that no benefit accrued from the additional help [120]. The enhanced programme involved further training in the use of LVAs and provision of alternative LVAs if necessary. It also incorporated advice on lighting and other features of the home environment during the course of a single home visit by a rehabilitation officer. The 'enhancement' may simply have been too little. For example, other work [121] indicated that standard low vision care followed by additional teaching sessions enabling extra tuition, correction of poor skills, additional practice with more difficult tasks and answering patients' questions over a 4 week period resulted in improvements in vision function (NEI-VFQ) and reading speed (Pepper VRST) compared with a group who had only standard care. Although there was no measurable improvement in VA, the experimental group reported improvements in self-rated eyesight. Whereas in the Reeves et al study [120] participants were given advice on lighting in the home, low vision centres in Sweden go further by upgrading lighting in the homes of patients (in kitchen, hall and bathroom) where necessary. A study to evaluate the benefits of improved lighting provision [122] indicated improvements in some ADL performance (mainly in kitchen and bathroom) with better lighting, although there was deterioration in other ADLs, possibly due to deterioration in vision during the study. As part of the study, half the participants were provided with optimum task and mood lighting in the living room (according to individual needs). The control group reported improvements in general health, self-confidence and loneliness (not from a published scale), but not to well-being (Psychological and General Well-being Index [123]) at 6 months after light adaptation. The intervention group showed significant improvements in the same items but also reported improved physical condition, appetite, social contacts, self-confidence, temper, depressed mood, vitality and well-being (not psychometrically evaluated measures). As the living room is a place associated with leisure, it may be that enhanced ability to enjoy valued leisure activities influenced perceptions of these constructs. This possibility chimes with evidence cited in section 3.1 that loss of valued activities is associated with increased depression. Other work suggested that increases in illuminance of task lighting leads to improvements in contrast sensitivity and near VA [124] and the authors suggested that provision of additional task illuminance should be part of low vision rehabilitation.

Training in eccentric viewing has been shown to improve reading speeds [125]. No PROs were used in this study, but other work reported here (e.g. [12], Mitchell, 2003) has indicated the impact of losing reading fluency due to MD and it is likely that there would be a positive impact on QoL for people trained in eccentric viewing or assisted by low vision aids to regain reading fluency.

### 6.2 Psychosocial interventions

Psychosocial interventions to assist adjustment to MD are not generally available in health-care settings [126] but a number of studies offer evidence for the benefits of such programmes. In Sweden a course of eight weekly 2-hour meetings for groups of 4–6 people with bilateral MD led by occupational therapists was evaluated initially by qualitative focus group methodology [90,127]. Participants' endorsement of their increased knowledge of MD, the social support offered by the meetings and improved self-efficacy in performing activities of daily living was supported by long-term evaluation of the course (28 months later) using quantitative methods [128]. Activities of daily living were investigated in the areas of meal preparation, self-care, communication, cleaning, mobility, shopping and financial management. Compared with people who had experienced standard care, those taking part in the courses reported higher levels of perceived security in performing 15 of 28 daily activities investigated including items relating to meal preparation, cleaning, mobility, communication and financial management. Self-care and shopping-related items were unaffected. Within the experimental group, participants reported improvement in 14 of 28 items at the 28 month evaluation, including items relating to meals, communication, cleaning, mobility and financial management. The control group reported lower levels of security in 12 of the 28 activities investigated, including items relating to meals, communication, cleaning, mobility, shopping and financial management. These findings illustrate the importance of education and support in preventing a decline in perceived security in performing ADLs as well as enabling greater perceived security.

A self-management intervention consisting of 6 weekly 2-hour group sessions was developed in the USA [129]. It included presentations, lectures, problem-solving and skills training with guided practice using cognitive-behavioural principles. Compared with a waiting list control group, participants reported improvements in emotional distress (POMS) and self-efficacy (scale developed for the study) and their use of LVAs almost doubled. Self-reported functional capacity and symptoms (QWB) did not improve. In a larger study using this programme, participants with higher baseline levels of depression reported greater improvements (POMS) compared with less depressed people in the experimental group and with depressed people in the control group. The experimental group showed improvements in vision function (NEI-VFQ) compared with controls (especially those more depressed at baseline). Improved self-efficacy was associated with decreased depression. The effects of the intervention were sustained at 6-month follow-up [130] when depression was lower overall in the experimental group than in the control group.

A cognitive-behavioural-based programme developed in Germany incorporated modules relating to relaxation, awareness of the associations between thought, emotion and behaviour, maximising personal and external resources, exchange of personal experience and provision of information [126]. In this small pilot study, the course of 5 weekly 2-hour sessions resulted in the experimental group (N = 14) reporting improvements in negative affect and depression (Positive and Negative Affect Schedule [131], GDS [115]), performance of ADLs (Multilevel Assessment Instrument [132], modified), personal autonomy (developed for study) and active problem orientation (Freiburg Inventory on Coping with Illness [133]) compared with the control group (N = 8).

In Leicester, UK, in a programme of peer support, 6 weekly 1 1/2-hour discussion groups were led by 3–4 people with at least 5 years' experience of living with MD [134]. Participants received leaflets which acted as guidelines for each topic for discussion in the group. Participant course evaluation indicated that the leaflets had given them useful information and that the aims of the course (to provide information about MD, to provide friendship and support and to help adjustment to MD) had been met. This was a small pilot study with 2 groups of 6 participants each. The sessions were well attended. Data indicated that people who reported poorest well-being (W-BQ12 [15]) at the start of the course showed significantly more improvement in well-being following the intervention. Thus greatest benefit was experienced by those most in need. Ten of the 11 participants completing questionnaires at follow-up said they would be willing to help run similar groups in the future for others newly diagnosed with MD, providing further evidence that the programme of peer support was beneficial for them and, with their assistance as future course leaders, could be rolled out to others.

### 6.3 Levels of rehabilitation and support

Evidence shows that rehabilitation offers benefits to functional status and a number of psychological outcomes. Nevertheless, the services offered are patchy. A survey of 588 blind and partially sighted older people (with eye conditions including MD) in the UK [135] reported poor levels of support and care, with only 53% of participants having had a home assessment, around half receiving help with household tasks and shopping and as few as 7% receiving meals on wheels. Many lived isolated lives, with 62% living alone and 11% receiving a visit from a trusted person less than once a month. It was estimated that 82% of those who lived alone were in poverty. Although all the people in the survey were eligible for registration as partially sighted or blind, only 58% knew of a local society for visually impaired people. There was a similar picture of inadequate provision of low vision aids and equipment to help with cooking and housework. In a survey in West Glamorgan of people registered blind or partially sighted [84], of 66 participants (39 with MD) only 80% had been visited by a social worker. Few possessed safety or mobility aids although all those who had them found them helpful. Kitchen aids, tactiles (e.g. raised buttons stuck on to oven controls to indicate temperature settings), and talking timepieces were also owned by only a minority. Distribution of aids was irregular with some owning several and others none at all. Many people were not aware of the financial benefits that might be available to them.

Clearly there are many improvements that could be made to rehabilitation services for people with MD. Although none of the rehabilitation studies reviewed here measured the effect of interventions on QoL, many reported improvements in outcomes that may contribute to QoL.

## 7. Medical treatment

In the Sub-Foveal Radiotherapy Study [19], the small clinical benefits of treatment of sub-foveal choroidal neovascularisation were not reflected in improvements to visual functioning (DLTV) or health status (SF-36) in a longitudinal analysis of a randomised clinical trial involving 199 participants (99 treatment, 100 control).

Submacular surgery trials initially used the SF-36 health status measure as the only PRO in the pilot study [20]. Changes in VA over the two year period of the study were not associated with changes in SF-36 scores. In the main trial the SF-36 was used again together with the Hospital Anxiety and Depression Scale (HADS) and NEI-VFQ. At enrolment, VA was associated with NEI-VFQ scores but not with HADS or SF-36 scores [28]. At follow-up [21] (2 years after enrolment) there were no significant changes in any of the PRO scores although participants in the treatment group were less likely to lose ≥ 6 lines of VA than the control group.

In a study of 50 patients who underwent macular translocation [24], pre-and post-operative visual function (NEI-VFQ) and general health status (SF-12) were measured. Overall vision function (NEI-VFQ) improved post-operatively and specifically in the subscales measuring general vision, difficulty with distance vision tasks, difficulty with near vision tasks, dependency, mental health and social function. Improvement in near and distance VA was associated with greater improvement in function (NEI-VFQ). Reading speed improved for 29 patients and this was associated with improvements in NEI-VFQ scores. Not surprisingly, there were no changes to SF-12 scores. Other work has reported improvements in reading speed following macular translocation [136,137].

Photodynamic therapy (PDT) for the treatment of sub-foveal neovascular MD has undergone extensive trials, but there has been little effort to measure its effect on QoL or any other PROs that may influence QoL. In one prospective study of PDT [138], at 12 months follow-up, 34 of 48 treated patients had lost fewer than 3 lines of VA and, on average, the sample lost fewer than two lines. VF-14 items showed either no change or a decrease in function, consistent with the VA changes. Participants reported being less anxious because of MD and that they stayed indoors less, but this may have been due to adjusting to living with MD. There was no control group in this study.

An unusual attempt to assess the impact of PDT on so called 'QoL', was based on a computer-generated model [56]. Hypothetical patients (characterised using data from the TAP study [139]) were the 'participants'. Utility values, acquired using TTO, were obtained by the researchers from Canadian patients with MD (N = 40) and utility values were calculated for a range of VA values. Utilities associated with loss or non-loss of three lines of vision over two years were calculated and the authors incorporated 'disutilities' judged to be associated with complications of PDT (determined only by ophthalmologists). The model was used to estimate QALYs gained as a result of PDT. Sharma et al calculated that a gain in QALYs of 11.3% could be expected from PDT. Sharma et al misleadingly used the terms 'QALYs' and 'utilities' interchangeably with 'QoL' suggesting that they were unaware of the poor relationship between TTO measures and measures of the impact of MD on QoL [52]. TTO and SG measures favoured by health economists are so far removed from patients' experience of the impact of MD on their QoL that it is a major source of concern that such studies may be used by NICE to determine which, if any, patients with MD will receive treatment on the National Health Service. NICE has taken the view that taxpayers, rather than patients, should be involved in decisions concerning the allocation of public funds for medical treatments and, in some determinations of utility values, panels made up of members of the public are used to elicit data [51]. Panel members are given descriptions of the medical conditions or health states and asked to evaluate their utility. Other exponents of value-based medicine do at least recommend that the utility values obtained from people who have the condition concerned should be used in preference to utility values estimated by surrogates [140].

Trials of new treatments, using anti-VEGF drugs to prevent the growth of new blood vessels characteristic of wet MD, have been promising. In a study of ranibizumab (Lucentis) in people with minimally classic or occult MD, treatment groups showed significant improvements in NEI-VFQ scores (near and distance activities and dependency) over one year compared with controls [141]. A one-year study of the effects of pegaptanib sodium (Macugen) compared with sham treatment [142] showed improvements in some NEI-VFQ subscale scores in the Macugen treated groups compared with controls but other subscales showed no change, possibly due to small subgroup sizes. Both of these abstracts refer inappropriately to QoL while actually reporting visual function. However, the results certainly look promising for QoL (providing that the experience of treatment with intraocular injections is not too distressing) even though QoL has yet to be measured.

A small study (30 intervention,15 controls) evaluated the effect of carotenoids and antioxidant supplementation in people with intermediate or advanced MD [143]. After one year, people with intermediate MD showed stabilized VA, better VA than controls and improved NEI-VFQ 39 visual function scores, while these deteriorated in the control group. There was no benefit found in the advanced MD group.

Several of these trials show promising results in terms of visual function but, while some have claimed to measure the impact of treatment on QoL, none has actually done so.

## 8. Future research

A diagnosis of MD will inevitably impact not only on patients but on those people closest to them. There may be concerns about genetic transmission of the condition to sons and daughters. A partner or other family member is likely to experience anxiety about the diagnosis and prognosis. Worry about the future and the patient's possibly deteriorating vision will affect those closest to him or her. Retired couples often enjoy shared interests and, where these are lost to the patient, they may also be lost to the partner. This may lead to resentment and frustration. There is also the possibility that people will over-compensate for the difficulties brought about by MD and make the person with MD unnecessarily dependent, preventing successful adjustment to the condition. People who develop MD are sometimes carers themselves and an inability to continue effectively in this role can lead to difficulties [144]. The cared-for person may then have to depend on more formal care, with the expense and disruption that it involves. People with MD may themselves have to be cared for. This sometimes entails moving in with their family and it is unlikely to be without consequences. There appears to have been no research carried out into this aspect of living with MD and findings from such studies may be helpful in showing how people with MD can most effectively be supported.

Although there has been research into LVA provision and training, only one study was found that reported investigation of the usefulness of specific devices [145]. The study reported that, in general, conventional optical aids were preferable to head-mounted devices for older users. However, participants did not select the devices they tried but were randomised to two of four devices. It may have been more informative if people had been able to choose which devices they tried, so overcoming any antipathy towards a particular device. More research is needed into the benefits of aids such as closed circuit television, portable electronic magnifying devices and voice-activated computer software.

In the UK, several hospital eye clinics make arrangements for volunteers with MD to set up a hospital volunteer support service to provide immediate information and support for people newly diagnosed with MD. Anecdotal evidence suggests that these services have been valuable and much appreciated by those who have been able to talk to volunteers and make contact with local MD groups and LVA services. A carefully designed evaluation study of the existing hospital volunteer services is needed, including clinics with and without such services, and further evaluation following introduction of new volunteer services where there were none previously. The MacSSQ measure of satisfaction with the service provided has been designed for such a study and may supply compelling evidence to encourage hospital eye clinics to be more open to such innovations, providing much needed initial help and support to bewildered patients. Peer support groups such as those pilot tested in Leicester [134], recruiting via hospital volunteers, also warrant evaluation in larger scale intervention programmes.

With new treatments being developed there is a need for treatment satisfaction to be evaluated. While possible new treatments for MD are welcome, patients may experience difficulties with them in a number of ways including side effects, apprehension, discomfort or distress caused by the method of administration, frequency of treatment and efficacy of treatment. Investigating patient satisfaction with treatment can lead to improvements that make treatments more acceptable to patients [146]. Foundations have been laid to develop an MD treatment satisfaction questionnaire, following the procedure used in the design of the RetTSQ measure of satisfaction with treatment for diabetic retinopathy [146].

## Discussion

Quality of life has been conceptualised in a number of ways in research into the impact of MD. Well-being, health status, functional status and visual function have all been referred to more or less inappropriately as QoL. We have argued that the use of such measures can be misleading, resulting in misinterpretation of findings. Nevertheless, PRO instruments other than QoL measures provide valuable data and, together, a variety of types of measure can give a fuller picture of the effect of MD on people's lives. Some measures, such as health status questionnaires are clearly not helpful in evaluating the effects of MD. The SF-36 is a comprehensively validated and widely used measure and it may be that it is selected purely on the grounds of its ubiquity. If it is not expected to yield relevant results, however, it is an unnecessary burden on participants and is likely to give an underestimation of the benefits of an intervention designed to improve vision. In any study or clinical trial, careful thought must be given to the choice of measures to ensure that the data collected are the data that are required to answer the research question or investigate the effect of an intervention. A treatment for MD may result in enhanced visual function but, if the treatment is very unpleasant, has to be repeated regularly and is anticipated with trepidation by some patients and refused by others, then it might do substantial damage to QoL in spite of having the potential to improve visual function. The work reported here indicates that, in many cases, there is widespread confusion about the term 'quality of life' and, generally, the choice of questionnaire indicates that it is defined inadequately. Choice of PRO instrument notwithstanding, measuring the impact of vision impairment is complicated by the involvement of a second eye and the interactions between the two eyes' visual status [12].

Utility measures are increasingly used to estimate so called 'QoL' gains or losses. The QALY values obtained using TTO and SG methods are not measuring QoL [12] and such measures give no impression of the ways in which MD impacts on people's lives. There are many reasons why a person may not want to relinquish any years of life in spite of serious visual impairment but this does not imply that they are content with the present situation or that their QoL would not be much better without their vision problems. When such measures are obtained from members of the public who have no awareness of living with MD the results are so far removed from the patients' experiences as to be completely irrelevant to QoL measurement. These inadequate and inappropriate measures and others like them have been the preferred instruments for 'QoL' measurement and continue to be used uncritically in organisations such as the UK's NICE [26], dominated by health economists who are committed to such inferential methods of measurement, unaware of the importance of psychological factors and unaccustomed to listening to patients' accounts of their own experiences and descriptions of the impact of MD on their lives.

What emerges from the work reviewed here is that MD has a damaging effect on many aspects of people's lives. The loss of central vision associated with MD impairs important aspects of visual function including reading, driving, recognising faces, watching TV and other near vision activities. Impaired visual function affects different people in different ways. Not all aspects of impairment will be important to all people with MD but evidence from studies using the MacDQoL shows that loss of visual function will affect all people with MD in some way [42,44]. The extent to which MD impacts QoL will be influenced by individual lifestyles and personal characteristics as well as by factors such as social support, co-morbidity and access to related services including training in the use of low vision aids.

A good deal of evidence indicates that poor psychological well-being and depression are more prevalent in people with MD than in the population at large. Depression has been associated with loss of valued activities [74] and with poor experiences at diagnosis, including being told that 'nothing can be done' [75]. Effective low vision rehabilitation [122] and self-management training [130] have been shown to assuage poor psychological well-being. Although promising treatments are being developed, findings about their impact on PROs are limited and we have yet to hear of any treatment demonstrating benefits to QoL using a genuine measure of QoL. It is hoped that future trials will go beyond measuring visual function and will include recently developed individualised measures of QoL [42,44]. Given that there is no cure available for anyone with MD and treatment to stabilise the condition is limited to a small proportion of people with wet MD, there is an urgent need to find other ways to curtail the damage to QoL caused by MD.

A greater public awareness of MD and the consequences of developing the condition are important. At present, levels of awareness are strikingly low [147], with the highest, 30%, in the USA. In some countries with a prevalence of MD comparable with the USA (e.g. Netherlands, Spain, Italy) fewer than 10% of the population are aware of the condition [147]. Increased awareness would lead to people consulting health professionals earlier and being diagnosed with MD in time for any treatment that may be appropriate to be effective in preserving vision. Evidence suggests that the affective quality of diagnostic consultations can have a long-term impact on patients' well-being (Mitchell, 2003). Good provision of information about MD, about its consequences and ways in which patients can monitor their vision and prevent unnecessary damage is important [89]. Information about support groups such as the AMD Alliance and the UK MD Society and about other organisations involved in helping visually impaired people may improve patient satisfaction with their experience of diagnosis and may improve their long-term well-being. Effective support and referral to other services as needed is important for well-being. The widespread provision of effective rehabilitation services is essential for the reduction of mental health problems which may develop into clinical depression. Rehabilitation treatment needs to be given as soon as possible and be designed to suit individual needs [12]. Evaluation of MD patients' experiences of services provided using purpose-designed instruments such as the MacSSQ [89] will identify deficiencies and enable targeted improvements to be made to the services provided. Use of an appropriate QoL measure, such as the MacDQoL will inform clinicians about the impact of MD on individuals' QoL and help in identifying priorities in the planning of rehabilitation programmes. Professional psychological or psychiatric support should be offered when needed. Well-being measures such as the W-BQ12 [15], a measure that has been evaluated for use with people with MD, can be valuable in screening for unusually high levels of depression and anxiety.

The use of QoL, well-being and vision function measures in assessing the value of changes to the services offered to people with MD will help to ensure that management of this group of patients is effective. Slakter and Stur [12] asserted that, ideally, different trials should use the same measures to enable comparison of the effects. The evidence cited in this review attests to the confusion that arises out of using a multiplicity of measures, some of which are insensitive and many of which are used inappropriately. It would be premature to recommend a specific set of measures for use in all trials, as some of the instruments are relatively new, though it is clear that health status measures such as the SF-36 and EQ5D are of little relevance and utility values derived using TTO and SG methods are misleading and best avoided. A variety of visual function measures are available, with the NEI-VFQ [38] being well established as a useful measure of visual function in MD and commonly used in clinical trials [24,28,142]. There is considerable evidence for the value of the W-BQ12 measure of well-being (e.g [148-150]), now psychometrically evaluated for people with MD [15]. There is growing evidence for the usefulness of the relatively recent MacDQoL measure of the impact of MD on quality of life which was developed specifically for people with MD [42,44,151]. Such measures of well-being and quality of life are urgently needed in clinical trials in addition to measures of visual function in a context of continuing evaluation of their sensitivity to change in response to treatments and rehabilitation for MD.

Data from the MDSQ [75] indicate that, before diagnosis with MD, many older people lead active and fulfilling lives, enjoying good quality of life and contributing to the quality of life of others. There are promising early indications that heroic medical and surgical interventions may prevent or even reverse the effects of MD for the small proportion of patients affected by specific forms of wet MD. These developments are very welcome. Nevertheless, we cannot wait for a cure for MD to restore good quality of life to all people with MD. Solutions to many of the problems highlighted in this report, such as improved empathy in diagnostic consultations, provision of good information, support and advice on self-help, would cost little or nothing to implement. The potential to improve the QoL of all people with MD exists, despite there being no cure, if there is the will to invest in rehabilitation, including low vision aid provision and training and in ongoing support. Urgent action is needed worldwide to:

• increase public awareness

• improve training for health professionals in communication skills and use and interpretation of PRO measures

• increase the provision of support, rehabilitation and LVA services

• evaluate services provided with audit which includes PRO measurement

• increase use of appropriate QoL measures as well as other suitable PRO measures in clinical trials of new treatments

• increase funding for:

◦ research

◦ implementation of research findings

◦ continuing evaluation of the impact of diagnosis and service provision on visual function, well-being, satisfaction and quality of life of people with MD

## Conclusion

Macular degeneration has a profoundly negative effect on people's lives. Much could be done to improve outcomes. Increased awareness of MD and its effects is needed together with efforts to improve the experience of those who are diagnosed with MD, through better information and support, improved facilities for rehabilitation and continuing evaluation of interventions using measures of quality of life, patient satisfaction and psychological well-being as well as measures of vision. With the very real promise of a rapid increase in the incidence of MD, action is needed now to protect and improve outcomes for people with MD including the most important outcome of all, their quality of life.

## Abbreviations

ADL activities of daily living

ADVS Activities of Daily Vision Scale

AMD Age-related macular degeneration

CDS Community Disability Scale

CNV choroidal neovascularisation

DLTV Daily Tasks Dependent on Vision Scale

DSM IV Diagnostic and Statistical Manual of Mental Disorders

EQ5D 5-item EuroQol questionnaire

FS functional status

FVSQ Functional Vision Screening Questionnaire

GDS Geriatric Depression Scale

GHQ General Health Questionnaire

HADS Hospital Anxiety and Depression Scale

HS health status

HUI-3 health utility index, version 3

IADL Instrumental Activities of Daily Living scale

LVA Low vision aid

LVQOL Low Vision Quality of Life measure

MacDQoL Macular Disease-Dependent Quality of Life measure

MD macular degeneration

MDSQ Macular Disease Society Questionnaire

MLVQ Manchester Low Vision Questionnaire

NEI-VFQ National Eye Institute Vision Function Questionnaire

NICE National Institute for Health and Clinical Excellence

PDT Photodynamic therapy

POMS Profile of Mood States

PRO patient reported outcome

QALY quality of life year

QoL quality of life

QWB Quality of Well-being Scale

SF-36 Short form 36

SG standard gamble

SIP Sickness Impact Profile

SIPV Sickness Impact Profile (vision)

TTO time trade-off

VA visual acuity

VF visual function

VF-14 14-item Visual Function scale

W-BQ12 12-item Well-being Questionnaire

## Declaration of possible conflicts of interest

The authors have jointly been involved in the design and/or development of the following questionnaires referred to in this review: MacSSQ, W-BQ12, and MacDQoL and are planning to undertake design work on a MacTSQ to measure satisfaction with treatments for MD. CB has also been involved in the design of the ADDQoL, RetDQoL and RetTSQ also mentioned here. Copyright in all these measures is owned by CB who licenses them to her company, Health Psychology Research Ltd, which draws up licence agreements for users and charges licence fees to commercial companies. For further information contact Professor Clare Bradley.

## Authors' contributions

CB and JM planned the literature search which was conducted by JM. JM wrote the first draft of the review. CB and JM jointly refined the manuscript and incorporated suggestions from those who commented on the early draft. Both authors approved the final version of the paper.
